# Glycine and N-acetylcysteine supplementation, with or without exercise, in brain health and functional aging: implications for sarcopenia and frailty in older adults

**DOI:** 10.3389/fnut.2026.1775264

**Published:** 2026-05-18

**Authors:** Xiaolan Wang, Ruiliang Hou, Zhihao Chen, Xiaoyang Wang, Haoyu Wang

**Affiliations:** 1 Medical College, Xijing University, Xi'an, Shaanxi, China; 2 Kuala Lumpur University of Science and Technology, Kuala Lumpur, Malaysia; 3 Department of Orthopedics, The Second Affiliated Hospital, Xi'an Jiaotong University, Xi'an, Shaanxi, China

**Keywords:** aging, chronic inflammation, exercise, glycine, GlyNAC, N-acetylcysteine, oxidative stress, sarcopenia

## Abstract

Aging is closely associated with oxidative stress, mitochondrial dysfunction, chronic inflammation, and progressive declines in muscle and cognitive function. Exercise is widely recognized as the most effective non-pharmacological strategy to counteract these processes; however, its benefits may be potentiated by targeted nutritional interventions. Glycine and N-acetylcysteine (NAC), both precursors of the antioxidant glutathione, have emerged as promising candidates for maintaining redox balance, supporting mitochondrial metabolism, and improving physiological resilience in older adults. Evidence on NAC suggests context-dependent effects, with supplementation improving glutathione availability, fatigue resistance, and exercise performance in individuals with low baseline glutathione, while results remain inconsistent in healthy populations. Glycine and its derivatives, such as glycine propionyl-L-carnitine, show potential to enhance anaerobic performance and reduce lactate accumulation, though findings are mixed and require confirmation in older cohorts. Increasingly, studies on combined glycine and NAC supplementation (GlyNAC) provide compelling proof-of-concept: clinical and preclinical trials demonstrate improvements in oxidative stress, mitochondrial dysfunction, insulin resistance, inflammation, muscle strength, cognition, and even lifespan extension in animal models. These results suggest that GlyNAC, especially when paired with exercise, may represent a novel paradigm to mitigate aging hallmarks and extend healthspan.

## 1 Introduction

Aging is characterized by progressive physiological decline, including loss of skeletal muscle mass and function (Sarcopenia), reduced mitochondrial efficiency, and increased oxidative stress, all of which contribute to frailty and diminished quality of life in older adults (1). In this review, the term “older adults” generally refers to individuals aged ≥60 or ≥65 years, consistent with definitions used by the World Health Organization and most geriatric clinical trials. However, the specific age range varies slightly across cited studies, and we report the original study definitions when relevant.

By the age of 70, individuals may experience a 25%−30% reduction in muscle mass, which is strongly associated with impaired mobility, falls, and loss of independence (1). In parallel, mitochondrial dysfunction and an imbalance between reactive oxygen species (ROS) production and antioxidant defense systems accelerate cellular senescence and contribute to age-related metabolic disorders (2). Exercise remains the most effective non-pharmacological intervention to counteract these changes. Both aerobic and resistance training improve muscle strength, enhance mitochondrial biogenesis, and reduce systemic inflammation in older populations (3). However, the adaptive response to exercise is often blunted in aging due to chronic low-grade inflammation, impaired redox balance, and limited substrate availability (4, 5). These observations have prompted growing interest in nutritional strategies, particularly amino acid supplementation, as adjuncts to exercise for maintaining health and function in aging.

Amino acids play multifaceted roles beyond protein synthesis. Glycine, a conditionally essential amino acid, is critical for glutathione synthesis, collagen stability, and mitochondrial function (6). Low circulating glycine levels have been associated with insulin resistance, obesity, and higher cardiometabolic risk (7, 8). Experimental studies suggest glycine supplementation may improve antioxidant defense, reduce inflammation, and support metabolic resilience, though direct trials in older adults remain scarce (9, 10). Derivatives such as glycine-propionyl-L-carnitine (GPLC) have been explored for their ergogenic potential, enhancing nitric oxide bioavailability and modulating exercise metabolism (11). N-acetylcysteine (NAC), another amino acid derivative, is a precursor of glutathione and a well-established antioxidant (12). In exercise physiology, NAC has been shown to mitigate oxidative stress, delay fatigue, and improve muscle redox status, particularly in individuals with low baseline glutathione levels (13). While short-term NAC supplementation may enhance performance and reduce oxidative damage, some evidence also suggests potential pro-oxidant effects under inflammatory conditions, highlighting the need for context-specific application (14, 15).

The synergy between amino acid supplementation and exercise may be particularly relevant in aging. By reducing exercise-induced oxidative stress, improving mitochondrial redox balance, and supporting protein synthesis, amino acids such as glycine and NAC could amplify the benefits of physical training. This integrative approach aligns with the broader goal of developing safe, accessible, and non-pharmacological strategies to delay functional decline in older adults. However, clinical evidence remains limited, with heterogeneous study designs and inconsistent findings. Thus, the objective of this review is to critically evaluate current evidence regarding glycine, N-acetylcysteine (NAC), and their combined formulation (GlyNAC) in the context of aging. Specifically, we aim to:

(1) summarize mechanistic pathways linking these amino acids to redox balance, mitochondrial function, and inflammation;
(2) examine clinical evidence regarding exercise performance, sarcopenia, metabolic health, and cognitive outcomes in older adults; and
(3) identify limitations, safety considerations, and research gaps for future investigation.

## 2 Search strategy and data sources

To comprehensively investigate the current literature surrounding this topic, a structured narrative review was conducted. An exhaustive literature search was executed across three primary scientific databases: PubMed/MEDLINE, Scopus, and Web of Science. The search strategy utilized a combination of Medical Subject Headings (MeSH) and specific free-text keywords, interconnected via Boolean operators (AND/OR) to maximize search sensitivity. The primary search algorithm incorporated the following terms: “glycine,” “N-acetylcysteine,” “GlyNAC,” “exercise,” “aging,” “sarcopenia,” “mitochondrial dysfunction,” and “oxidative stress.” To ensure a contemporary and highly relevant scope, the temporal parameters for the initial literature retrieval were restricted to peer-reviewed articles published between January 2000 and 2025. Furthermore, supplementary backward citation tracking was performed manually by screening the reference lists of retrieved seminal articles and previous systematic reviews to identify any additional pertinent literature that may have eluded the primary database search.

The inclusion criteria were deliberately formulated to capture both observable clinical outcomes and their underlying physiological mechanisms. We prioritized evidence derived from human clinical trials, placing a distinct emphasis on randomized controlled trials (RCTs) and comprehensive systematic reviews published from 2015 onwards, reflecting the most recent and rigorous advancements in the field. To establish a robust mechanistic framework—particularly concerning mitochondrial bioenergetics and redox homeostasis, highly relevant *in vivo* and *in vitro* preclinical models were also deemed eligible for inclusion.

Articles were excluded if they lacked peer review or were available only as abstracts without full-text access. The screening process was conducted iteratively; it began with a meticulous evaluation of article titles and abstracts to determine preliminary relevance, followed by an in-depth full-text appraisal. Throughout this selection process, utmost priority was explicitly assigned to studies evaluating targeted interventions in older adult populations experiencing sarcopenia or age-associated functional decline. Extracted data from the final selection of articles were then narratively synthesized to delineate the current state of knowledge and identify existing gaps in the literature.

## 3 Exercise as a modulator of aging

**Figure 1:**
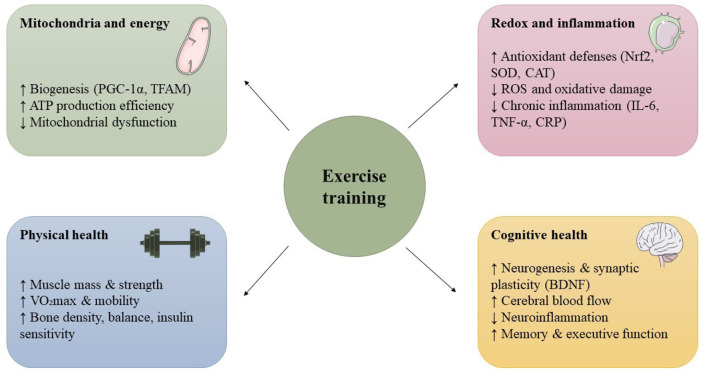
Multisystem effects of exercise on biological aging pathways. Exercise as a modulator of aging. Regular physical activity supports healthier aging by enhancing mitochondrial function, strengthening antioxidant defenses, reducing chronic inflammation, and preserving muscle, cardiovascular, and brain health. While adaptations may be blunted in older adults due to frailty and reduced regenerative capacity, exercise remains one of the most effective strategies to slow age-related decline.

Regular exercise is widely recognized as a powerful modulator of the aging process (16). Aging is strongly influenced by lifestyle factors. Among these, physical activity stands out as a safe, cost-effective way to improve health outcomes in older adults (16). Through its broad effects on cells and organs, exercise can help slow age-related decline. For example, consistent training enhances mitochondrial function, strengthens antioxidant defenses, dampens chronic inflammation, and preserves muscle, cardiovascular and brain health. As illustrated in Figure 1, exercise modulates multiple biological pathways implicated in aging.

### 3.1 Mitochondrial effects of exercise

Aging is associated with a loss of mitochondrial capacity: muscle and other tissues tend to have fewer, smaller, and less efficient mitochondria as we grow older. Exercise counteracts this decline by stimulating mitochondrial biogenesis and quality control. Endurance training activates key regulators such as PGC-1α, NRF1 and TFAM, leading to the production of new, functional mitochondria. In one study, 12 weeks of aerobic exercise in older rats restored PGC-1α and TFAM levels to above those of young untrained controls. Importantly, human trials mirror this finding: months of regular endurance exercise in sedentary seniors increased muscle mitochondrial DNA, oxidative enzyme activities (citrate synthase, COX) and electron transport chain function (17).

These adaptations can be quite robust. For instance, one trial reported that 16 weeks of moderate aerobic training raised mitochondrial volume density by ~50% in previously inactive older adults, with concomitant increases in PGC-1α and TFAM expression (18). Such data suggest that the “mitochondrial aging” seen in elders is largely due to sedentary lifestyle rather than inevitable aging. Indeed, when older subjects exercised regularly, their mitochondria resembled those of young people (17). In some cases, exercise even improves mitochondrial efficiency: specialized training protocols (like high-intensity or resistance exercise) have been shown to boost ATP production per mitochondrion, reducing wasteful electron leakage (17). In short, habitual exercise preserves or restores mitochondrial number, structure and function in aged muscle. By maintaining energetic capacity and coupling efficiency, training helps older adults meet energy demands for movement and cellular repair.

### 3.2 Oxidative stress and inflammation

One hallmark of aging is chronic oxidative stress and low-grade inflammation. Exercise has a dual but ultimately beneficial effect on these processes. Each workout produces an acute burst of reactive oxygen species (ROS) in muscle, which paradoxically triggers the cell's defense mechanisms a phenomenon known as mitohormesis (17). In particular, exercise-induced ROS activate the redox-sensitive transcription factor Nrf2 and other antioxidants, ramping up the body's own capacity to neutralize free radicals (17). Over time this adaptive response reduces steady-state oxidative damage. For example, animal studies report that regular endurance exercise in aged rodents lowers markers of oxidative DNA damage (8-oxo-dG) in muscle and increases DNA repair enzymes (17). At the same time, chronic training tends to lower basal oxidative damage and inflammation. Long-term exercise training has been shown to reduce age-related oxidative damage and chronic inflammation (19). In humans, sedentary elders who began moderate-intensity programs often exhibit lower levels of circulating oxidative stress markers after a few months, even if classic antioxidant enzyme levels do not change much (17). Exercise also modulates the immune system: repeated bouts of activity create an overall anti-inflammatory environment (20). For instance, trained older adults have lower resting levels of pro-inflammatory cytokines (IL-6, TNFα, CRP) and higher IL-10 than untrained peers (20). In aggregate, these training effects counteract the “inflamm-aging” trend. Regular exercise thus helps reset the redox balance and inflammation. By bolstering antioxidant defenses and shifting cytokine profiles, exercise slows the usual age-related rise in oxidative stress and inflammatory signaling (19, 21). In practical terms, this means reduced tissue damage, better vascular function, and a lower risk of inflammation-related diseases in active seniors.

### 3.3 Physical capacity and metabolic health

The systemic benefits of exercise in older adults extend to muscle strength, endurance, and metabolism. Resistance (strength) training in elders reliably increases muscle mass and power, and aerobic training preserves aerobic fitness and mitochondrial enzymes. In meta-analyses, older men and women (even into their 80s) showed significant gains in lean mass and strength after 12+ weeks of high-volume, moderate-to-heavy resistance exercise (22, 23). These improvements boost mobility and function in daily life. Aerobic activities (walking, cycling, etc.) likewise improve VO_2_max and walking speed, and sustain oxidative enzyme levels in muscle (24, 25). Notably, trained older individuals can achieve levels of muscle oxidative capacity comparable to much younger exercisers, despite smaller muscle fiber size (17). Metabolically, exercise training enhances insulin sensitivity and growth-factor signaling. Chronic exercise upregulates the IGF-1 pathway and improves glucose uptake in muscle (19). It also lowers blood lipids and body fat, improving overall metabolic profile (21). These changes reduce age-related risks like type 2 diabetes and atherosclerosis. In effect, active seniors tend to have healthier body composition, better balance and coordination, and preserved bone density—all contributing to independence. For example, one study notes that even frail octogenarians can gain muscle (albeit less than younger people) with tailored high-intensity training (26). The key is that exercise interventions in elders often combine resistance, aerobic, flexibility and balance work, each targeting different functional domains (19).

### 3.4 Cognitive and brain health

Aging brains benefit from physical activity, too. Exercise promotes neuronal plasticity, especially in memory-related regions. In animal models, running increases neurogenesis in the hippocampus and angiogenesis in cortex, improving oxygen and nutrient delivery. Human neuroimaging studies mirror this: fitter older adults tend to have larger hippocampal and prefrontal volumes and better cognitive test scores (21, 27, 28). Crucially, interventional trials suggest these brain benefits are real. In one landmark RCT, about 120 sedentary seniors were randomized to 12 months of moderate walking vs. stretching. The walkers showed a significant increase in hippocampal volume over 1 year (whereas controls did not), and this change correlated with better spatial memory and higher BDNF levels (29). Exercise also improves brain vascular health and reduces neuroinflammation. Regular training helps maintain endothelial function and cerebral blood flow, while dampening oxidative stress and inflammatory processes in brain vessels (30). These effects may protect against age-related cognitive decline. In fact, systematic reviews find that older adults who engage in regular aerobic and resistance training often enjoy modest improvements in attention, executive function and memory compared to sedentary peers. While results vary, the consensus is that sustained moderate exercise builds “cognitive reserve” meaning the brain's resilience to aging in the elderly (31, 32).

### 3.5 Challenges: blunted adaptations and frailty

Despite these benefits, older adults face some limitations. A well-documented issue is anabolic resistance: aged muscle has a reduced protein synthesis response to exercise. In practice, identical training yields smaller hypertrophy and strength gains in seniors than in young adults (33–35). For example, one study found men and women over 80 had blunted muscle-fiber growth compared to younger trainees, even with the same program (36, 37). This blunted adaptation means that higher or more prolonged stimulus (longer training periods, optimal nutrition) is often needed to achieve gains in elders. Frailty adds another challenge. Frail seniors have low reserve and multiple system impairments, making intense exercise risky or impractical. By definition, frailty compromises strength, balance, and metabolism (19). Accordingly, exercise programs must be carefully tailored. Experts recommend a multicomponent approach for frail elders: combining strength, aerobic, flexibility and balance exercises, adjusted to the individual's capacity (19). Progress must be gradual, and supervision or physical therapy support is often needed. Even so, studies show that with proper guidance, very old or frail individuals can make functional gains. One review notes that even in the oldest old (80+ years), structured resistance training over 12+ weeks leads to significant improvements in muscle mass and function (38, 39). Overall, exercise exerts broad anti-aging effects at molecular and systemic levels (19, 21). While aging blunts the magnitude of these adaptations, the direction is the same: active older adults consistently show healthier physiology than their sedentary peers. Tailored exercise prescriptions, often combined with good nutrition, remain one of the most powerful tools to mitigate age-related decline.

## 4 Amino acid supplementation and exercise in aging

### 4.1 Glycine in aging and exercise-related physiology

Glycine, the simplest amino acid, plays a key role in metabolic regulation, collagen synthesis, and antioxidative defense. Its relevance to exercise and aging has recently attracted attention, as glycine availability declines with age, potentially contributing to impaired muscle repair, reduced metabolic flexibility, and increased oxidative stress (40). Evidence suggests that circulating glycine levels are linked to metabolic health in older adults. In a study of functionally limited older adults, higher serum glycine was associated with lower insulin resistance and reduced central adiposity, highlighting its potential role in maintaining glucose homeostasis and body composition (41). This metabolic regulation may indirectly influence exercise tolerance and recovery, especially in populations at risk for Sarcopenia and frailty.

Clinical studies examining direct supplementation are limited, but glycine's involvement in glutathione synthesis suggests a protective effect against exercise-induced oxidative stress. By enhancing intracellular antioxidant capacity, glycine could theoretically improve muscle recovery following intense or unaccustomed activity (6). Moreover, its role in collagen and creatine synthesis supports structural integrity and energy metabolism, both essential for maintaining functional capacity during aging (42). Although direct trials on glycine alone in older adults undergoing exercise training are scarce, studies with glycine derivatives (e.g., glycine-propionyl-L-carnitine, discussed in Section 4.2) show potential benefits in enhancing exercise performance and reducing lactate accumulation. This provides indirect evidence that glycine-related pathways may support exercise adaptation in aging populations (43). In summary, glycine appears to contribute to healthy aging by supporting antioxidant defenses, metabolic health, and musculoskeletal function. However, robust randomized controlled trials specifically assessing glycine supplementation with exercise in older adults are lacking, making this an area ripe for future investigation.

### 4.2 Glycine derivatives: glycine propionyl-L-carnitine (GPLC)

Glycine propionyl-L-carnitine (GPLC) has attracted interest as a dietary supplement due to its potential to increase nitric oxide (NO) bioavailability and improve exercise performance. Acute supplementation appears to transiently enhance performance under certain conditions, while longer-term use shows less consistent effects. Evidence from a randomized, double-blind, placebo-controlled, cross-over study in 24 resistance-trained males found that a single 4.5 g dose of GPLC significantly improved peak and mean power output during repeated Wingate sprints. Across five bouts, sprint performance increased by 2.6%−15% compared with placebo. Additionally, blood lactate levels were 15.7% lower 4 min post-exercise (*P* = 0.09) and 16.2% lower 14 min post-exercise (*P* < 0.05), suggesting improved lactate clearance and reduced metabolic stress. These findings indicate that GPLC may acutely support high-intensity performance by enhancing NO availability and buffering lactate accumulation (11). In contrast, an 8-week supplementation trial that combined GPLC with endurance training in healthy men and women reported no ergogenic benefit. Participants received either placebo, 1 g/day GPLC, or 3 g/day GPLC alongside a structured aerobic training program. At the end of the intervention, there were no significant changes in muscle carnitine levels, peak oxygen uptake (VO_2_peak), time to fatigue, anaerobic threshold, or total work across groups (*P* > 0.05). These results suggest that chronic GPLC use, even at higher doses, does not meaningfully alter muscle carnitine storage or improve aerobic or anaerobic performance in recreationally active individuals (44).

### 4.3 Other amino acids and peptides with exercise in aging

Beyond glycine and its derivatives, several amino acids and peptides have been studied for their potential to enhance exercise adaptations and counteract age-related declines in muscle function and health. As shown in Table 1, soy peptides have been evaluated in combination with exercise as a functional food intervention for older adults. In a randomized controlled trial of 72 community-dwelling older Japanese adults, participants were assigned to exercise with or without soy peptide supplementation for 3 months. While both groups improved muscle mass index by 2%−3%, only the supplementation group showed a significant improvement in memory function (+0.3 points on cognitive testing), suggesting an additive benefit of soy peptides on cognitive domains alongside exercise (45). Citrulline (CIT) and leucine (LEU), both known stimulators of protein synthesis, have been investigated in older women with low BMI. In a 20-week double-blind trial, women who combined weekly exercise with CIT·LEU supplementation (0.8 g CIT + 1.6 g LEU twice daily) demonstrated significant increases in body weight, BMI, muscle mass, and physical activity levels compared to placebo (*P* < 0.05). These findings suggest that targeted amino acid supplementation can help address Sarcopenia and frailty in at-risk older populations (46).

**Table 1:** Summary of clinical trials investigating amino acid supplementation combined with exercise in older adults.

Population/model	Design and intervention	Exercise type	Key findings	References
16 men (69 ± 3 years), 15 women (68 ± 4 years)	Randomized trial; whey protein (WP) or collagen peptides (CP), 30 g twice daily, during energy restriction + step reduction + recovery	No structured exercise during ER/SR; habitual activity resumed in recovery	WP, but not CP, enhanced lean mass and muscle protein synthesis recovery after inactivity; protein did not prevent lean mass loss during restriction.	(48)
72 community-dwelling older adults	RCT; 3-month exercise + cognitive training, with or without weekly soy peptide supplementation	Multicomponent exercise and cognitive training, 1 × /week	Soy + exercise improved memory scores; both groups improved skeletal muscle mass index, but no extra muscle benefit from soy.	(45)
Elderly women (mean 76.7 years)	Double-blind RCT; HMB (2 g), arginine (5 g), lysine (1.5 g) daily for 12 weeks	No structured exercise (general activity in elderly)	Supplementation improved functionality, leg strength, handgrip strength, and protein synthesis; positive trend in fat-free mass.	(47)
Older Japanese women with low BMI (16–21 kg/m^2^)	RCT; citrulline (0.8 g) + leucine (1.6 g) twice daily for 20 weeks	Weekly weight-bearing + square stepping exercise (75 min)	CIT+LEU with exercise increased body weight, BMI, body mass, and physical activity; may help prevent sarcopenia and frailty.	(46)

Other studies have examined combinations of β-hydroxy-β-methylbutyrate (HMB), arginine, and lysine. In two parallel randomized, double-blind trials involving elderly women (mean age 76.7 years), supplementation for 12 weeks improved functionality (17% faster “get-up-and-go” test, *P* = 0.002), leg strength, handgrip, and limb circumference. Protein synthesis, assessed with 15N-glycine tracer, increased by ~20% compared to placebo (*P* = 0.03), highlighting the potential of this mixture to both stimulate anabolism and reduce protein breakdown in aging muscle (47). Protein quality also appears to influence recovery during periods of inactivity and energy restriction. In a controlled trial of 31 older adults (mean age ~69 years), whey protein (WP) and collagen peptide (CP) supplementation were compared during 3 weeks of reduced energy intake and step reduction. Both groups lost lean mass during inactivity, but only the WP group regained leg lean mass and enhanced muscle protein synthesis during recovery (*P* = 0.05). This underscores the importance of protein source, with whey demonstrating greater efficacy for muscle recovery compared to collagen (48). Collectively, these studies indicate that amino acid and peptide-based interventions can complement exercise in older adults, but their effects vary depending on the compound, dosage, and physiological context. Soy peptides may support cognition, CIT·LEU may prevent sarcopenia in underweight women, HMB-arginine-lysine mixtures enhance muscle function, and whey protein appears superior for recovery during inactivity.

### 4.4 Integrative perspective

Glycine represents one of several amino acids under investigation for their potential to enhance the benefits of exercise in aging populations. While direct clinical trials combining glycine supplementation with exercise in older adults are lacking, glycine's established metabolic functions provide a strong rationale for exploration. In particular, its role as a precursor for glutathione synthesis supports antioxidant defense, reducing oxidative stress that contributes to sarcopenia and metabolic decline. Across studies of other amino acids and peptides, several common mechanisms emerge: improved redox balance, enhanced mitochondrial metabolism, stimulation of muscle protein synthesis, and even cognitive benefits when paired with physical activity. For instance, leucine and citrulline supplementation promote anabolic signaling and muscle maintenance, while soy peptides combined with exercise have been linked to better memory outcomes.

Beyond its role as a glutathione precursor, glycine exerts independent mechanisms critical for mitigating sarcopenia. At the structural level, glycine is the most abundant amino acid in collagen, making it an indispensable substrate for maintaining the tensile strength of intramuscular connective tissue, fascia, and tendons, structures that are vital for mechanical force transmission and are often compromised in frail individuals. Metabolically, glycine supports continuous creatine synthesis via the enzyme glycine amidinotransferase, acting as a critical ATP buffer during skeletal muscle contraction. Furthermore, glycine demonstrates direct anti-inflammatory properties by inhibiting the production of pro-inflammatory cytokines (e.g., IL-6 and TNF-α) in macrophages, thereby helping to blunt the catabolic environment associated with aging (40, 49).

These parallels suggest that glycine, given its central role in one-carbon and energy metabolism, could similarly act as a synergistic adjunct to exercise in preserving function during aging. The current research gap lies in the absence of targeted randomized controlled trials (RCTs) evaluating glycine plus structured exercise interventions in older adults. Existing evidence on glycine derivatives, such as glycine propionyl-L-carnitine, supports its potential for enhancing exercise capacity and lowering lactate accumulation, but translation to aging cohorts remains unexplored. Considering glycine's safety profile, affordability, and biological plausibility, future trials are warranted to determine its efficacy as a complementary strategy to exercise for mitigating age-related decline in muscle and cognitive health.

## 5 N-acetylcysteine (NAC) and exercise in aging

### 5.1 Mechanistic rationale for NAC in exercise and aging

**Figure 2:**
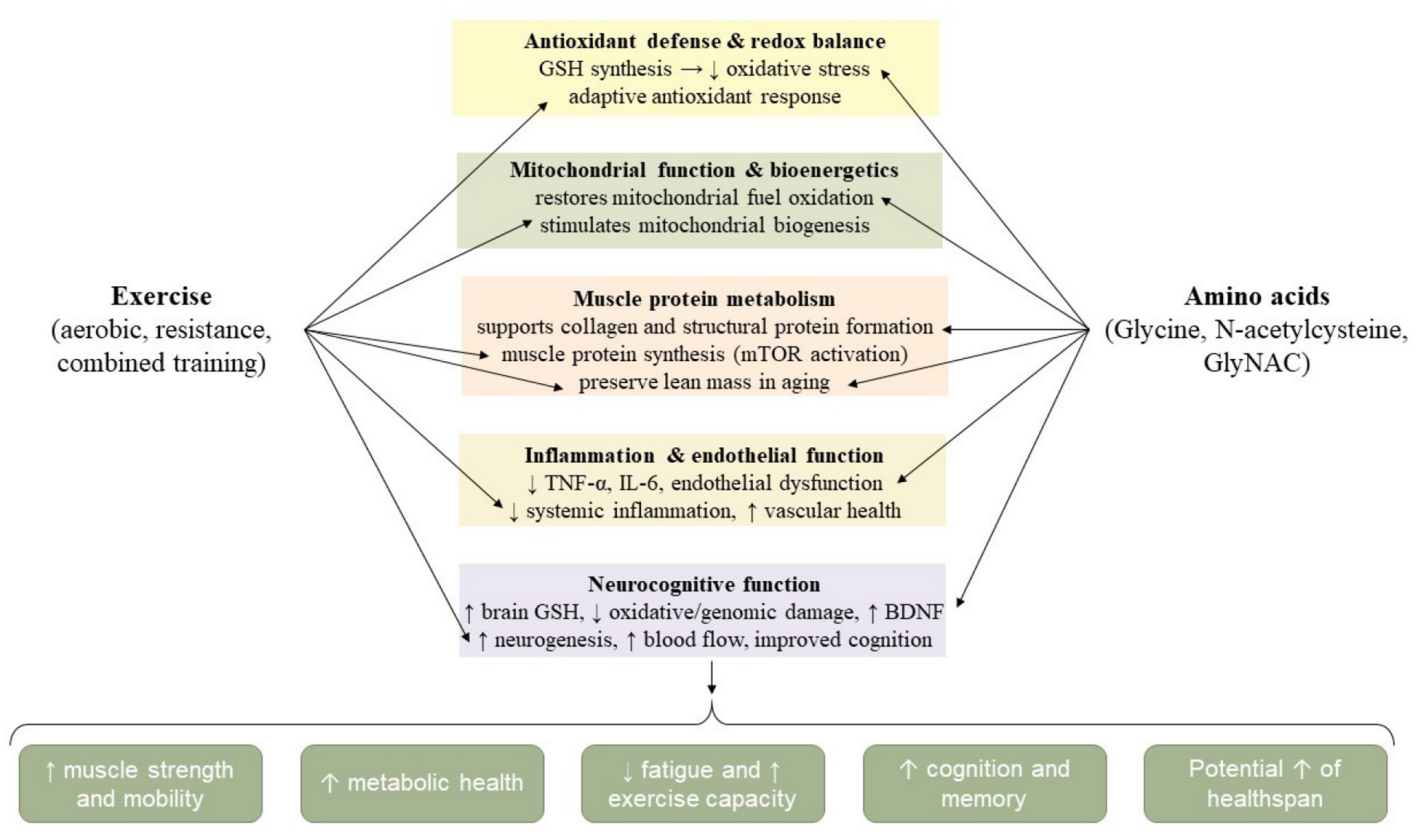
Mechanisms of N-acetylcysteine (NAC) in redox regulation during exercise and aging. Integrative model of exercise and nutritional strategies in aging. The figure illustrates how exercise (aerobic, resistance, or combined) and amino acid-based interventions (glycine, N-acetylcysteine, or their combination as GlyNAC) act through overlapping biological pathways. These include enhanced antioxidant defenses and redox balance, improved mitochondrial function and bioenergetics, stimulation of muscle protein metabolism, reduction of inflammation and endothelial dysfunction, and support of neurocognitive health. Together, these mechanisms translate into improved muscle strength, mobility, metabolic health, exercise capacity, and cognition, ultimately contributing to healthier aging and extended healthspan.

Intense or unaccustomed exercise increases the production of reactive oxygen species (ROS), which can overwhelm the antioxidant defenses of the cell and contribute to oxidative stress. This imbalance impairs cellular function, disrupts DNA integrity, and alters redox-sensitive signaling pathways involved in gene expression. Persistent oxidative stress has been implicated in multiple pathological states, including cancer, Parkinson's disease, and Alzheimer's disease. As illustrated in Figure 2, N-acetylcysteine (NAC) functions as a cysteine donor that supports intracellular glutathione synthesis. By supporting GSH synthesis, NAC helps maintain redox balance and prevents lipid peroxidation of cellular membranes, which is commonly exacerbated by exercise-induced oxidative stress. Thus, NAC offers a mechanistic basis for protecting cells against the detrimental effects of ROS generated during physical activity, particularly in older adults who already exhibit diminished antioxidant capacity (50). However, the antioxidant role of NAC is not universally protective, and under certain inflammatory conditions it may even act as a pro-oxidant.

In the context of aging muscle, the primary mechanistic value of NAC lies in its ability to protect mitochondrial bioenergetics. Sarcopenia is characterized by increased mitochondrial ROS leakage and reduced ATP production. By replenishing intracellular cysteine, NAC preserves the mitochondrial membrane potential and shields the electron transport chain (ETC) from oxidative degradation. This targeted reduction in mitochondrial ROS prevents the downstream activation of apoptotic and catabolic pathways that drive muscle fiber atrophy (50, 51).

As shown in Table 2, in a human study using an eccentric exercise model to induce acute muscle injury, supplementation with NAC (10 mg/kg/day) combined with vitamin C transiently increased oxidative damage markers, including lipid hydroperoxides and 8-iso-prostaglandin F2α. Muscle damage biomarkers such as lactate dehydrogenase and creatine kinase were also elevated to a greater extent in the antioxidant group compared to placebo. These findings suggest that during periods of heightened inflammation, NAC, particularly when paired with other antioxidants, may paradoxically exacerbate oxidative stress and tissue injury. This underscores the importance of considering exercise context, dosage, and baseline redox status when applying NAC as an adjunct in exercise interventions, especially in aging populations where inflammation and oxidative stress are already elevated (14).

**Table 2:** Effects of N-Acetylcysteine (NAC) supplementation on exercise, oxidative stress, and recovery.

Population/model	Design and intervention	Exercise type	Key findings	References
36 healthy adults, stratified by glutathione (low/mod/high)	Double-blind crossover; NAC 2 × 600 mg twice daily × 30 days	VO_2_max, time trial, Wingate	NAC improved performance (+13%−15%) only in low-glutathione group; restored GSH, reduced oxidative stress	(53)
29 young adults (21.3 ± 4 years)	Randomized, 21 days NAC vs. placebo	High-intensity eccentric elbow flexion (80% 1RM)	All groups: ↑ soreness and oxidative damage; NAC groups maintained IL-10 longer, suggesting prolonged anti-inflammatory response	(59)
Healthy men, eccentric arm muscle injury model	Placebo vs. NAC+Vit C (7 days, acute)	Acute eccentric-induced muscle injury	NAC+Vit C group had higher oxidative stress (lipid hydroperoxides, 8-iso-PGF2α), more muscle damage markers	(14)
Eight endurance-trained men	Double-blind crossover; IV NAC infusion (125 mg·kg^−1^·h^−1^ then 25 mg·kg^−1^·h^−1^)	Prolonged cycling (71% VO_2_peak, then to fatigue at 92% VO_2_peak)	NAC ↑ time to fatigue by 26%; ↑ muscle cysteine and GSH availability	(54)
Healthy adults, handgrip tests	Oral NAC (150 mg·kg^−1^) vs. placebo	Isometric and repetitive submaximal handgrip	NAC delayed fatigue (~130% baseline) and inhibited glutathione oxidation	(55)
17 semi-elite male rugby players	RCT, oral NAC 1 g/day × 6 days	Broken bronco sprint test	Mixed: ↓ soreness days 1–4, ↑ soreness days 5–6; slight sprint benefit, no consistent performance gain	(56)
Eight men, heavy exercise protocol	Acute oral NAC 1,800 mg vs. placebo	30-min heavy constant load test	NAC reduced respiratory muscle fatigue (higher PImax)	(57)
Healthy adults	Oral NAC capsules (9–18 mg/kg) or solution (35–140 mg/kg)	Handgrip exercise	NAC ↑ plasma cysteine and glutathione in dose-dependent manner; 70 mg/kg optimal for thiol status	(58)

### 5.2 Effects on exercise performance and fatigue resistance

Evidence from controlled trials and systematic reviews indicates that NAC supplementation has the potential to improve exercise performance by supporting glutathione homeostasis and reducing oxidative stress. A recent systematic review of 16 controlled trials in adult men reported that NAC supplementation was generally safe and associated with improved exercise outcomes, including enhanced antioxidant capacity and endurance performance. However, the review also noted variability in responses, with limited benefits observed in inflammatory and hematological markers, suggesting that the ergogenic effects of NAC may be context-dependent (52). Inter-individual variability in response to NAC appears to be influenced by baseline glutathione levels. In a double-blind crossover trial, individuals with low resting glutathione showed marked improvements in performance metrics following 30 days of NAC supplementation (2 × 600 mg, twice daily). VO_2_max increased by 13.6%, time-trial performance improved by 15.4%, and Wingate anaerobic capacity rose by 11.4%. These effects were absent in participants with normal or high baseline glutathione, highlighting that NAC may be most beneficial in individuals with redox deficiencies (53). At the muscular level, NAC has been shown to delay fatigue in endurance-trained athletes. In a double-blind crossover study, intravenous NAC infusion extended time-to-fatigue at 92% VO_2_peak by 26% (6.4 ± 0.6 vs. 5.3 ± 0.7 min; *P* < 0.05). This improvement was accompanied by elevated muscle cysteine and glutathione availability, suggesting that NAC's fatigue-resisting effects are mediated by improved redox buffering in active skeletal muscle (54). Similar results were observed in localized muscle tasks. In a handgrip fatigue protocol, oral NAC administration (150 mg·kg^−1^) increased repetitive submaximal task endurance by 30% compared to baseline, while placebo showed only a 15% increase, likely due to a training effect. NAC supplementation was also associated with reduced glutathione oxidation, providing further evidence that the attenuation of oxidative stress contributes to delayed fatigue (55). In high-intensity team sports, NAC's effects appear less consistent. A study in semi-elite rugby players found that 6 days of oral NAC supplementation (1 g/day) produced a likely beneficial effect on maximal sprint performance (2.4% improvement) and reduced perceived muscle soreness during the initial days of supplementation. However, muscle soreness increased after repeated bouts of intense exercise, suggesting that NAC may confer short-term recovery benefits but could have diminishing or even negative effects with prolonged use (56).

NAC has also been investigated for its effects on respiratory muscle fatigue during heavy exercise. In a trial involving oral NAC (1,800 mg) given 45 min before exercise, participants showed a preservation of inspiratory muscle strength compared to placebo, where maximal inspiratory pressure dropped by ~14% after 30 min. This indicates that NAC may reduce respiratory muscle fatigue, a known limiting factor in high-intensity endurance tasks (57). Dose-response studies further suggest that NAC's ergogenic benefits are linked to its thiol-donor activity, but higher doses increase the risk of gastrointestinal side effects. Oral doses of 70 mg/kg were shown to significantly elevate plasma cysteine and glutathione levels without major adverse effects, whereas 140 mg/kg caused significant gastrointestinal discomfort. These findings indicate that moderate dosing can optimize redox balance and performance without compromising tolerability (58).

### 5.3 Influence on oxidative stress and redox status

Exercise is a potent stimulator of oxidative stress due to the increased generation of reactive oxygen species (ROS). While moderate ROS signaling is essential for adaptation, excessive oxidative stress can impair DNA integrity, cellular function, and recovery. NAC, as a cysteine donor and glutathione precursor, plays a critical role in maintaining redox balance and protecting against lipid peroxidation. Its potential to support antioxidant defenses during exercise has driven interest in NAC as a supplement to mitigate oxidative stress in both athletic and aging populations (50). In a randomized trial, 29 young adults supplemented with NAC for 21 days demonstrated altered responses to eccentric exercise-induced oxidative stress. Although all groups exhibited increases in markers of oxidative damage, such as malondialdehyde and protein carbonyls, NAC supplementation was associated with higher levels of the anti-inflammatory cytokine IL-10 up to 7 days post-exercise. This suggests that NAC may modulate the inflammatory response to muscle-damaging exercise, supporting recovery through enhanced antioxidant and cytokine regulation rather than completely eliminating oxidative stress (59). However, evidence also indicates that NAC can act as a pro-oxidant under certain conditions. In a study inducing acute muscle injury via eccentric contractions, participants receiving NAC (10 mg/kg/day) together with vitamin C showed higher levels of lipid hydroperoxides and 8-iso-PGF2α compared to placebo. These individuals also exhibited greater elevations in lactate dehydrogenase and creatine kinase, markers of muscle damage. The findings suggest that excessive antioxidant supplementation during acute inflammatory states may paradoxically worsen oxidative stress and tissue injury (14).

At the muscular level, NAC's influence on redox status has been demonstrated in endurance-trained athletes. Intravenous NAC infusion during prolonged cycling increased both muscle total and reduced glutathione concentrations compared to placebo, while also elevating cysteine and cystine availability (*P* < 0.001). Importantly, NAC prevented the exercise-induced decline in muscle glutathione and reduced the rise in oxidized glutathione observed in controls. These effects translated into enhanced fatigue resistance, providing mechanistic support for NAC's role in maintaining redox balance during intense exercise (55).

### 5.4 Inflammatory response and recovery

NAC supplementation appears to influence post-exercise inflammatory responses, particularly after eccentric muscle-damaging activity. In a randomized study of 29 young adults, participants who received NAC for 21 days showed altered cytokine responses following high-intensity eccentric exercise. While all groups experienced elevations in muscle soreness, lipid peroxidation, and protein carbonyls after exercise, only NAC-supplemented individuals maintained higher levels of the anti-inflammatory cytokine IL-10 by day seven. This suggests that NAC may support recovery by promoting sustained anti-inflammatory signaling, even though it did not fully prevent oxidative damage or soreness (59). In contrast, other evidence points to potential risks when NAC is consumed immediately after acute injury. In an eccentric muscle injury model, individuals supplemented with NAC (10 mg/kg/day) and vitamin C for 7 days exhibited greater elevations in muscle damage markers such as creatine kinase and lactate dehydrogenase, alongside higher levels of lipid hydroperoxides and 8-iso-prostaglandin F2α compared to placebo. These findings indicate that NAC, particularly when combined with other antioxidants, may act as a pro-oxidant under acute inflammatory conditions, exacerbating tissue injury rather than alleviating it (14). In clinical populations, NAC combined with structured exercise has shown more consistent benefits. In patients with chronic obstructive pulmonary disease (COPD), a 12-week program of aerobic, resistance, and tai chi exercise with daily NAC supplementation (1,800 mg) significantly enhanced antioxidant defenses. Compared to placebo, the exercise-plus-NAC group demonstrated higher catalase (+25.4%), superoxide dismutase (+22.4%, *P* = 0.01), and glutathione peroxidase (+18.9%, *P* = 0.01) activity, along with a 26% reduction in malondialdehyde (*P* = 0.002), a marker of lipid peroxidation. These improvements suggest that NAC, when combined with chronic exercise, may optimize redox and inflammatory balance, aiding long-term recovery and adaptation (60).

### 5.5 Safety, adverse effects, and implications for older adults

Evidence on the safety profile of N-acetylcysteine (NAC) in the context of exercise is mixed. Most trials report NAC as generally well-tolerated, even at relatively high doses, with no serious adverse events (52, 58). However, gastrointestinal side effects such as nausea, bloating, and discomfort are dose-dependent and more frequent when intake exceeds 70 mg/kg orally (58). At these doses, NAC effectively elevates plasma cysteine and glutathione without severe intolerance, but higher levels (e.g., 140 mg/kg) markedly increase adverse reactions. Another limitation lies in the potential for NAC to act paradoxically as a pro-oxidant under certain conditions. For instance, in acute muscle injury models, NAC (often combined with vitamin C) amplified oxidative stress markers and worsened indices of muscle damage, suggesting that timing and context of supplementation are critical (14). This pro-oxidant shift may stem from interactions with free iron during the inflammatory phase. Thus, indiscriminate or immediate post-injury use of NAC may be counterproductive. Despite these concerns, NAC shows promise when used strategically, especially in populations with impaired redox balance. Older adults frequently experience glutathione deficiency, oxidative stress, and inflammation, all of which contribute to muscle fatigue, frailty, and reduced recovery capacity. In this context, NAC supplementation, particularly when combined with structured exercise, may restore redox homeostasis, reduce fatigue, and support healthier aging trajectories (53, 54, 60). Still, most available studies are small, short in duration, and often limited to younger or athletic populations, with few trials conducted directly in older adults. In summary, NAC supplementation is safe for most individuals at moderate doses and may enhance exercise performance, delay fatigue, and support recovery, especially in those with compromised antioxidant defenses. However, its benefits are less consistent in healthy, redox-replete individuals and may even be harmful under acute inflammatory stress. Larger, longer-term randomized trials in older populations are needed to confirm its role as a targeted adjunct to exercise for mitigating age-related oxidative stress and functional decline. Overall, the use of NAC in older adults should be individualized. Supplementation may be most appropriate in individuals with documented glutathione deficiency, elevated oxidative stress, or reduced exercise tolerance. Conversely, in the context of acute inflammation, uncontrolled chronic inflammatory conditions, or immediately following muscle injury, high-dose antioxidant supplementation should be approached cautiously, as excessive redox suppression may interfere with physiological adaptation or even exacerbate oxidative damage. Future studies should clarify optimal timing, dosage, and patient selection criteria to maximize benefits while minimizing potential risks.

## 6 Clinical evidence of GlyNAC supplementation

### 6.1 Synergistic molecular mechanisms counteracting sarcopenia and frailty

To fully appreciate the therapeutic efficacy of GlyNAC combined with exercise in older adults, it is imperative to delineate how this combination counteracts the interconnected molecular drivers of sarcopenia: oxidative stress, mitochondrial dysfunction, inflammaging, and anabolic resistance.

Overcoming the Dual Precursor Bottleneck: Aging muscle suffers from a severe depletion of intracellular glutathione (GSH). While cysteine (provided by NAC) is widely recognized as the rate-limiting precursor for GSH synthesis, advanced aging also precipitates a secondary bottleneck via systemic glycine deficiency. GlyNAC supplementation uniquely overcomes this dual limitation, maximizing the rate of GSH synthesis. This robust antioxidant defense neutralizes ROS, significantly reducing lipid peroxidation and protein carbonylation, which otherwise directly damage contractile proteins (10, 61).

Reversal of Mitochondrial Dysfunction: Frailty is intricately linked to a decline in mitochondrial quality control. By restoring homeostatic GSH levels, GlyNAC protects the mitochondrial organelle from self-inflicted oxidative damage. This synergy not only improves respiratory capacity and ATP output but also facilitates proper mitophagy, clearing dysfunctional mitochondria that accumulate in aged skeletal muscle (62). Suppression of “Inflammaging” and Catabolism: Chronic, low-grade inflammation in older adults activates the NF-κB signaling pathway. This activation subsequently upregulates muscle-specific E3 ubiquitin ligases, specifically *Atrogin-1* and *MuRF1*, which accelerate the degradation of muscle proteins. By systemically quenching oxidative stress and leveraging glycine's suppression of circulating cytokines, GlyNAC effectively blunts NF-κB activation, shifting the muscle microenvironment from a catabolic state to an anabolic one (63, 64).

Mitigation of Anabolic Resistance: Aging induces an “anabolic resistance,” wherein skeletal muscle becomes less responsive to the stimulatory effects of mechanical loading (exercise) and protein ingestion. The antioxidant shield provided by GlyNAC prevents ROS-induced interference with the mechanistic target of rapamycin (mTOR) signaling pathway. By protecting mTOR activation post-exercise, GlyNAC facilitates a more robust and efficient muscle protein synthesis (MPS) response, providing a clear molecular rationale for the significant improvements in handgrip strength, gait speed, and physical performance observed in clinical trials (61, 65).

### 6.2 Improvements in glutathione and redox status

GSH is the cell's primary antioxidant, essential for maintaining redox balance and limiting oxidative damage. Aging is consistently associated with a decline in glutathione defenses and an increase in oxidative stress (OxS), which contributes to mitochondrial dysfunction and multiple age-related pathologies. Given that glycine and cysteine are rate-limiting precursors for glutathione synthesis, supplementation with GlyNAC (glycine plus N-acetylcysteine) has been proposed as a strategy to restore GSH levels and redox balance in older adults. As shown in Table 3, randomized clinical trials have demonstrated that older adults exhibit lower reduced-to-oxidized glutathione ratios (GSH:GSSG) compared with young individuals, alongside elevated markers of oxidative stress. In one large placebo-controlled trial involving 114 healthy older adults (mean age 65 years), baseline oxidative stress markers were significantly elevated compared with a younger cohort (malondialdehyde 0.158 vs. 0.136 μmol/L, *P* < 0.0001; oxidized glutathione 174.5 vs. 132.3 μmol/L, *P* < 0.0001). Despite these abnormalities, GlyNAC supplementation for 2 weeks at doses ranging from 2.4 g to 7.2 g/day did not significantly increase total glutathione concentrations or the GSH:GSSG ratio in the overall cohort. However, *post-hoc* analysis revealed that participants with both high oxidative stress and low baseline GSH showed a significant rise in glutathione generation (905.4 vs. 819.7 mg/L for medium/high-dose GlyNAC vs. placebo, *P* = 0.016), suggesting that supplementation may particularly benefit subgroups with elevated redox demand (66, 67).

**Table 3:** Evidence from human and animal studies on GlyNAC supplementation and aging outcomes.

Study type	Population/ model	Sample size	Intervention	Key findings	References
RCT (2 weeks)	Healthy older adults (mean age 65)	114 OA + 20 young controls	GlyNAC at 2.4, 4.8 g, or 7.2 g/day vs. placebo	Safe and well-tolerated. No significant effect in full cohort, but *post-hoc* showed ↑ GSH in those with high oxidative stress and low baseline GSH (*P* = 0.016).	(66)
RCT (16 weeks)	Older adults (*n* = 24) + young adults (*n* = 12)	OA randomized to GlyNAC (12) or placebo (12); YA received GlyNAC	GlyNAC vs. alanine placebo	GlyNAC (not placebo) corrected GSH deficiency, ↓ oxidative stress, improved mitochondrial and endothelial function, ↓ insulin resistance, improved gait speed & strength.	(61)
Pilot (14 days)	Adults with type 2 diabetes	10 T2D + 10 non-diabetic controls	GlyNAC supplementation (14 days)	GlyNAC improved MFO (+30%, *P* < 0.001), ↓ glucose oxidation (−47%, *P* < 0.01), ↓ IR (−22%, *P* < 0.01), ↓ FFA (−25%, *P* < 0.01).	(69)
Open-label (36 weeks)	Older adults (*n* = 8) + young adults (*n* = 8)	OA supplemented for 24 weeks, then 12-week withdrawal	GlyNAC (dose not specified)	24-week GlyNAC improved GSH, ↓ oxidative stress, ↓ insulin resistance, ↓ inflammation, ↑ cognition, muscle strength, gait speed; effects declined after withdrawal.	(10)
Animal (lifespan)	C57BL/6J mice	Not reported	GlyNAC diet vs. control	GlyNAC ↑ lifespan by 24%, corrected GSH deficiency, ↓ oxidative stress, improved mitophagy and nutrient sensing.	(62)
Animal (8 weeks)	Old (90 weeks) and young (20 weeks) C57BL/6J mice	Not reported	GlyNAC diet vs. control	Old mice showed brain GSH deficiency, oxidative stress, mitochondrial dysfunction, inflammation, genomic damage, and cognitive decline; GlyNAC corrected these defects and reversed age-related cognitive decline.	(70)
Animal (7 weeks)	Old (30 months) mice	Not reported	NAC vs. NAC+Gly diet	NAC+Gly improved diastolic cardiac function, reduced left atrial volume and diastolic pressure, decreased leukocyte infiltration, upregulated mitochondrial genes, and improved ATP generation. NAC alone had no effect.	(63)
RCT (2 weeks)	Healthy older adults (mean age 65)	114 OA + 20 YA	GlyNAC at three doses (1.2/1.2, 2.4/2.4, 3.6/3.6 g)	No significant increase in total glutathione vs. placebo. Subgroup with high oxidative stress and low GSH showed increased glutathione. GlyNAC raised fasting glycine levels; in old mice, glycine supplementation improved mitochondrial respiration in skeletal muscle.	(67)
Animal (8 weeks)	Old (90 weeks) and young (20 weeks) mice	Not reported	GlyNAC diet vs. control	Old mice had brain GSH deficiency, oxidative stress, mitochondrial and glucose uptake defects, inflammation, and impaired cognition. GlyNAC corrected these abnormalities and improved maze performance.	(71)
RCT (16 weeks)	20 OA + 10 YA	OA randomized to GlyNAC vs. alanine; YA received GlyNAC	GlyNAC vs. placebo	In OA, glucose meal caused ↑ oxidative stress (+5.6%) and ↑ inflammation (+11.2% IL-6). GlyNAC supplementation protected against both (OxS −0.4%, IL-6 4.2%). No effect in placebo or YA.	(64)

Longer-duration interventions provide stronger evidence of efficacy. In a 16-week randomized controlled trial in 24 older adults, GlyNAC supplementation significantly corrected glutathione deficiency and lowered oxidative stress, while placebo had no effect. These improvements were accompanied by parallel benefits in mitochondrial function, inflammation, and endothelial health, underscoring the central role of glutathione restoration in broader metabolic resilience (2). Similarly, in a 36-week open-label trial, eight older adults showed normalization of red blood cell glutathione concentrations and marked reductions in oxidative stress after 24 weeks of supplementation. Importantly, when GlyNAC was withdrawn for 12 weeks, glutathione levels and redox markers reverted toward baseline (10). Additional clinical work has explored acute redox responses to metabolic stress. In a meal-challenge trial, older adults displayed exaggerated postprandial increases in oxidative stress (TBARS +5.6% vs. −0.1% in young adults, *P* < 0.0001) and inflammation (IL-6 +11.2 vs. +3.5, *P* < 0.0001) following glucose ingestion. Sixteen weeks of GlyNAC supplementation completely blunted this rise, reducing oxidative stress from +4.8% to −0.4% (*P* = 0.002) and lowering IL-6 response from +10.8 to +4.2 (*P* = 0.0001), while placebo showed no effect (64). This finding highlights GlyNAC's potential not only to restore basal glutathione levels but also to buffer oxidative insults triggered by everyday dietary challenges.

### 6.3 Effects on mitochondrial function and energy metabolism

Mitochondrial dysfunction is a central feature of aging and is tightly linked to impaired energy metabolism, oxidative stress, and functional decline in older adults. Multiple clinical studies have examined whether supplementation with glycine and N-acetylcysteine (GlyNAC), precursors of glutathione (GSH), can restore mitochondrial health and improve bioenergetics in this population. Evidence from human studies indicate that older adults consistently display reduced intracellular GSH, elevated oxidative stress, and impaired mitochondrial fatty acid oxidation compared to younger adults. In a randomized placebo-controlled trial involving 24 older adults and 12 younger controls, GlyNAC supplementation for 16 weeks significantly corrected GSH deficiency and improved mitochondrial function, while placebo had no effect. Improvements extended to molecular regulators of mitochondrial energy metabolism, with parallel reductions in insulin resistance and inflammation, suggesting that mitochondrial restoration translated into systemic metabolic benefits (61). A longer 36-week open-label study further reinforced these findings. Eight older adults received GlyNAC for 24 weeks followed by a 12-week withdrawal phase. Supplementation corrected GSH deficiency, restored mitochondrial fatty acid oxidation, and improved exercise capacity, muscle strength, and gait speed. Importantly, these benefits declined after discontinuation, highlighting the need for sustained supplementation to maintain mitochondrial and metabolic improvements (10).

Dose-finding trials in larger cohorts provide additional nuance. In a study of 114 healthy participants (mean age 65 years), GlyNAC given for 2 weeks at doses ranging from 1.2 to 3.6 g/day per amino acid did not significantly raise circulating GSH levels overall. However, subgroup analyses revealed that participants with higher oxidative stress and lower baseline GSH status responded with measurable increases in GSH and improved redox balance. Interestingly, even independent of GSH, supplementation restored fasting glycine levels, which are often reduced in aging. This finding is important because preclinical data suggest that glycine alone may enhance mitochondrial respiratory function, indicating that some of the mitochondrial benefits may be glycine-driven rather than entirely dependent on GSH synthesis (67). Another randomized trial examined GlyNAC's ability to blunt metabolic stress induced by a glucose challenge. After a 75 g oral glucose load, older adults exhibited significantly greater increases in oxidative stress (TBARS: +5.6 ± 0.6 vs. −0.1 ± 0.6 in young adults, *P* < 0.0001) and inflammation (IL-6: +11.2 ± 0.7 vs. +3.5 ± 1.2, *P* < 0.0001). Sixteen weeks of GlyNAC supplementation attenuated these postprandial mitochondrial and metabolic disturbances, while placebo showed no effect (10). Finally, a 36-week clinical study provided novel mechanistic insights into how GlyNAC affects brain energy metabolism. Older adults demonstrated a “brain glucose steal” phenomenon, where mitochondrial impairment in peripheral organs diverted glucose away from the brain. GlyNAC supplementation corrected mitochondrial dysfunction and reversed this imbalance, thereby improving brain glucose utilization and cognitive outcomes. These findings suggest a direct link between mitochondrial restoration, systemic energy distribution, and neurocognitive health (68). Complementary animal data strengthen the translational case for GlyNAC. In aged mice, GlyNAC supplementation restored GSH levels, reduced oxidative stress, and improved mitochondrial respiratory function in skeletal muscle. Notably, glycine supplementation alone was sufficient to enhance mitochondrial bioenergetics, supporting human trial data that glycine availability may independently modulate mitochondrial efficiency. Together, clinical and preclinical evidence demonstrates that improvements in mitochondrial function are consistently linked with better insulin sensitivity, muscle performance, and exercise capacity.

### 6.4 Reduction of systemic inflammation and endothelial dysfunction

Chronic low-grade inflammation and endothelial dysfunction are hallmarks of biological aging and are closely linked to cardiovascular risk, Sarcopenia, and frailty in older adults. These abnormalities worsen with age and may be improved by GlyNAC. Older adults consistently show higher circulating markers of inflammation and impaired endothelial function compared to younger populations. In a 16-week randomized placebo-controlled trial including 24 older and 12 younger adults, GlyNAC supplementation corrected intracellular GSH deficiency and significantly reduced systemic inflammation and endothelial dysfunction, while placebo treatment failed to produce any improvements. The intervention also led to parallel benefits in insulin resistance and oxidative stress, suggesting that restoration of redox balance may underlie the reduction in inflammatory activity (61).

A longer open-label trial extended these findings by evaluating 24 weeks of supplementation in eight older adults, followed by a 12-week withdrawal phase. GlyNAC intake lowered plasma inflammatory markers and improved indices of endothelial function, alongside reductions in oxidative stress, genomic damage, and insulin resistance. Importantly, these improvements declined after withdrawal, underscoring the necessity of continuous supplementation to sustain anti-inflammatory and vascular benefits (10). The effect of GlyNAC on meal-induced inflammation has also been tested. In a study of 20 older and 10 younger adults, a 75 g glucose load acutely triggered greater rises in oxidative stress (TBARS: +5.6 ± 0.6 vs. −0.1 ± 0.6, *P* < 0.0001) and inflammation (IL-6: +11.2 ± 0.7 vs. +3.5 ± 1.2, *P* < 0.0001) in older participants compared to young controls. After 16 weeks of GlyNAC, these exaggerated responses were markedly blunted, with TBARS falling from +4.8 ± 0.9 to −0.4 ± 0.8 (*P* = 0.002) and IL-6 from +10.8 ± 3.2 to +4.2 ± 2.3 (*P* = 0.0001). These effects were not observed in the placebo group, highlighting GlyNAC's protective role against postprandial inflammatory and endothelial stress (64).

Animal experiments provide additional mechanistic support. In aged mice, GlyNAC supplementation not only corrected GSH deficiency and oxidative stress but also lowered systemic inflammation and improved endothelial function. These effects coincided with restoration of mitochondrial fatty acid oxidation and reductions in insulin resistance, pointing toward an interconnected network in which redox balance governs vascular health and inflammatory tone (10). Together, clinical and preclinical findings strongly indicate that GlyNAC supplementation reduces systemic inflammation and improves endothelial function in older adults. Benefits are consistently observed across resting and postprandial states, suggesting that GlyNAC enhances both baseline and stress-induced vascular responses. Importantly, withdrawal of supplementation leads to loss of benefit, emphasizing the need for sustained intake. These findings provide compelling support for GlyNAC as a potential nutritional strategy to mitigate age-associated vascular dysfunction and systemic inflammation.

### 6.5 Impact on insulin resistance and metabolic health

Insulin resistance (IR) is a central feature of both aging and type 2 diabetes (T2D), often linked with mitochondrial dysfunction, oxidative stress, and altered substrate utilization. Evidence suggests that deficits in glutathione (GSH), the body's major endogenous antioxidant, contribute significantly to these abnormalities. Supplementation with glycine and N-acetylcysteine (GlyNAC), which provide the precursors needed for GSH synthesis, has been shown to improve mitochondrial function and metabolic health in human studies. In individuals with T2D, profound impairments in mitochondrial fuel handling and insulin sensitivity have been documented. Compared with non-diabetic controls, adults with T2D exhibited 36% lower mitochondrial fatty acid oxidation (MFO, *P* < 0.001), 106% higher mitochondrial glucose oxidation (MGO, *P* < 0.01), 425% greater insulin resistance (*P* < 0.001), and 76% higher plasma free fatty acids (FFA, *P* < 0.05). Following just 14 days of GlyNAC supplementation, significant metabolic improvements were observed: MFO increased by 30% (*P* < 0.001), MGO decreased by 47% (*P* < 0.01), IR was reduced by 22% (*P* < 0.01), and plasma FFA concentrations fell by 25% (*P* < 0.01). These findings highlight the potential of GlyNAC to restore more physiologic substrate oxidation, reduce lipotoxicity, and improve insulin sensitivity in T2D (69). Similar trends have been observed in older adults, where insulin resistance is strongly associated with oxidative stress, inflammation, and mitochondrial impairment. In a 36-week open-label trial, older participants received GlyNAC supplementation for 24 weeks, followed by a 12-week withdrawal phase. Supplementation led to robust improvements, including correction of intracellular GSH deficiency, reduction of oxidative stress, and restoration of mitochondrial function. Importantly, insulin resistance decreased during the supplementation period, along with improvements in other metabolic parameters such as body fat, waist circumference, and glucose handling. However, after discontinuation of GlyNAC, many of these benefits regressed, suggesting a continued requirement for supplementation to sustain metabolic improvements (10). Taken together, these changes translate into improved glucose handling and body composition, highlighting GlyNAC as a potential strategy for improving cardiometabolic health.

### 6.6 Cognitive and functional benefits

Cognitive decline and reduced physical function are common features of aging, often attributed to oxidative stress, mitochondrial dysfunction, and impaired metabolic homeostasis. Emerging evidence suggests that supplementation with GlyNAC (a combination of glycine and N-acetylcysteine) may target these underlying mechanisms and improve both brain and physical health in older adults. In a 36-week clinical trial comparing older adults (OAs) with young adults (YAs), baseline assessments revealed that OAs had significant deficits, including glutathione deficiency, elevated oxidative stress, mitochondrial dysfunction, inflammation, insulin resistance, and reduced levels of brain-derived neurotrophic factor (BDNF). These abnormalities were accompanied by measurable cognitive decline and impaired functional performance. Notably, OAs demonstrated slower gait speed, reduced grip strength, lower 6-min walk test capacity, and evidence of genomic damage, all reflecting hallmark features of age-related functional decline (10).

GlyNAC supplementation for 24 weeks led to broad improvements in both cognitive and physical domains. Improvements coincided with better redox and mitochondrial balance. Functional performance also improved: participants showed enhanced muscle strength, faster gait speed, and greater exercise capacity, alongside reductions in body fat and waist circumference. Importantly, these benefits diminished after GlyNAC withdrawal for 12 weeks, indicating that continuous supplementation may be required to sustain positive effects. A novel mechanistic insight from this study was the identification of a **“**brain glucose steal” phenomenon, whereby impaired mitochondrial function caused peripheral organs to utilize glucose at the expense of the brain. This redistribution of glucose was linked to reduced brain energy availability and cognitive impairment. Remarkably, GlyNAC supplementation reversed this phenomenon, thereby restoring brain glucose utilization and supporting improved cognitive performance (68). These pilot data suggest GlyNAC may enhance cognition and physical performance in aging.

### 6.7 Summary of clinical evidence and gaps

Current clinical studies investigating GlyNAC supplementation in older adults and individuals with type 2 diabetes provide promising proof-of-concept evidence. Across small pilot trials, GlyNAC has consistently demonstrated improvements in glutathione status, oxidative stress, mitochondrial function, insulin resistance, inflammation, cognitive performance, and physical capacity. These findings suggest that targeting fundamental aging hallmarks may translate into meaningful benefits for metabolic, cognitive, and functional health. Across domains, benefits were lost after discontinuation, suggesting the need for sustained supplementation. However, several important limitations must be acknowledged. Most published studies have been conducted in small cohorts, typically involving fewer than 20 participants, and over relatively short intervention periods ranging from 2 to 6 months. Additionally, nearly all clinical evidence to date originates from a single research group, raising the need for replication across independent centers and diverse populations. The absence of large-scale, RCTs limits the ability to draw definitive conclusions about efficacy, safety, and long-term outcomes. Future research should prioritize multicenter, adequately powered RCTs with longer follow-up periods to establish the durability of GlyNAC's effects and to determine its potential role in preventing or delaying age-related diseases. Moreover, dose–response relationships, adherence, and potential interactions with exercise or other interventions remain unexplored and warrant systematic evaluation. Addressing these gaps will be essential to move GlyNAC supplementation from early pilot findings toward broader clinical application. Despite promising findings, the GlyNAC literature remains limited by small sample sizes, short intervention durations, and reliance on surrogate biomarkers rather than hard clinical endpoints such as falls, hospitalization, or long-term functional decline. Furthermore, most studies originate from a single research group, highlighting the need for independent replication. While mechanistic improvements in redox status and mitochondrial function are compelling, their translation into sustained clinical benefit remains to be confirmed in larger trials.

## 7 Challenges and future perspectives

Despite growing interest in the interaction between amino acid supplementation and exercise in aging populations, several challenges remain before clear recommendations can be made. First, the heterogeneity of study designs, including differences in sample size, duration of interventions, types of exercise protocols, and dosages of supplements, makes it difficult to compare findings or establish standardized guidelines. Many clinical trials to date are limited by small cohorts (often < 30 participants) and short follow-up periods (typically 4–12 weeks), restricting conclusions about long-term efficacy and safety (1). Another challenge lies in population-specific responses. Older adults often present with comorbidities, polypharmacy, and nutritional deficiencies, all of which may influence the bioavailability and effectiveness of supplements such as glycine, NAC, or their derivatives (65). Moreover, variability in baseline redox status and muscle health appears to modulate the effect of antioxidant supplementation, as shown with NAC, which benefits individuals with low glutathione but may be ineffective, or even detrimental, in those with adequate levels (53). Importantly, antioxidant supplementation may attenuate some exercise-induced adaptive signaling pathways if administered inappropriately. Therefore, future research should distinguish between physiological redox signaling required for adaptation and pathological oxidative stress requiring intervention. Precision approaches based on baseline glutathione status, inflammatory markers, and frailty phenotype may help refine patient selection.

There are also methodological concerns. Many trials lack blinding, rely on subjective measures such as muscle soreness, or do not include gold-standard biomarkers of mitochondrial function, oxidative stress, and protein turnover. In addition, some antioxidant trials raise the concern of pro-oxidant effects under inflammatory conditions, suggesting that supplementation strategies may need to be carefully timed relative to exercise and recovery (14). From a translational perspective, combination approaches may hold the greatest promise. Emerging data suggest that amino acids such as glycine, leucine, or citrulline, when paired with structured exercise, can influence muscle metabolism, body composition, and even cognitive outcomes. However, direct RCTs testing glycine supplementation alongside exercise in older adults are still absent. Bridging this gap will require large-scale, multicenter studies with longer follow-up periods, standardized protocols, and clinically meaningful endpoints such as sarcopenia prevention, mobility preservation, and cognitive health. Looking forward, precision nutrition approaches may help identify which subgroups of older adults are most likely to benefit from specific amino acid interventions. Integrating metabolomics and redox profiling into future trials could allow personalization of supplementation strategies based on baseline deficiencies or metabolic signatures. If validated, such approaches could move amino acid supplementation from an experimental adjunct to an evidence-based component of healthy aging strategies.

## 8 Conclusions

The synthesis of clinical and preclinical evidence examining glycine and its derivatives, particularly Glycine Propionyl-L-Carnitine (GPLC), reveals a multifaceted role in exercise physiology and performance enhancement. This integrated analysis demonstrates that glycine supplementation operates through several interconnected mechanisms that collectively support athletic performance, recovery, and structural integrity. The primary mechanism of action centers on glycine's role as a precursor for glutathione synthesis, thereby enhancing intracellular antioxidant capacity and mitigating exercise-induced oxidative stress. This antioxidant effect represents a fundamental pathway through which glycine supplementation confers protective benefits during physical exertion, particularly in high-intensity and prolonged exercise modalities where reactive oxygen species production escalates significantly. Beyond its antioxidant properties, glycine contributes substantially to structural support mechanisms through collagen synthesis facilitation. This aspect proves particularly relevant for connective tissue integrity and muscle repair processes following mechanical stress during exercise. The evidence further indicates glycine's involvement in energy metabolism through creatine synthesis support, providing a mechanistic basis for improved ATP regeneration during anaerobic activities. Notably, GPLC demonstrates distinct lactate-buffering effects during high-intensity exercise, suggesting a unique role in modulating metabolic byproducts that contribute to fatigue. These mechanisms collectively operate through both direct biochemical pathways and indirect modulation of inflammatory responses, creating a comprehensive framework for understanding glycine's ergogenic potential.

Regarding dosage protocols, the evidence supports differentiated approaches based on specific performance objectives. For acute performance enhancement, a single 4.5 g dose of GPLC administered 30–60 min pre-exercise demonstrates efficacy in improving endurance parameters and reducing lactate accumulation. Chronic supplementation regimens typically involve 1–3 g daily divided doses, with optimal benefits emerging after a minimum 4-week loading period. The timing of administration proves critical, with pre-exercise dosing maximizing acute performance effects while post-exercise administration (within 30 min of completion) optimizes recovery processes through enhanced protein synthesis and reduced inflammatory markers. Special consideration must be given to population-specific factors, with older adults potentially requiring lower initial doses and gradual titration to achieve therapeutic benefits without adverse effects. The analysis of study designs reveals significant methodological heterogeneity that complicates direct comparisons across investigations. Clinical studies predominantly employ randomized controlled trial designs with crossover methodologies, focusing primarily on young to middle-aged athletic populations. These investigations typically assess outcomes through performance metrics (VO_2_max, power output), biochemical markers (lactate, oxidative stress indicators), and subjective recovery scores. Preclinical models, primarily utilizing rodent exercise paradigms, provide valuable mechanistic insights but suffer from translation limitations due to species differences. A consistent methodological gap identified across the literature involves the limited representation of older athletic populations in robust RCTs, creating an evidence void for this demographic that experiences age-related declines in glycine metabolism and antioxidant capacity.

Optimal exercise protocols for maximizing glycine supplementation benefits involve specific modalities and parameters. Resistance training at moderate to high intensities (70%−85% 1RM) with volume parameters of 3–5 sets of 8–12 repetitions demonstrates particular synergy with glycine's structural support mechanisms. Endurance exercise at 60%−75% VO_2_max for 30–60 min durations benefits from glycine's antioxidant and metabolic support properties. High-intensity interval training protocols, consisting of 4–8 intervals of 30–90 s at 85%−95% maximal effort, appear particularly responsive to GPLC's lactate-modulating effects. The evidence supports a minimum intervention period of 4 weeks for chronic adaptations, with ongoing supplementation recommended during periods of intensified training to maintain benefits. Administration strategies should be tailored to specific performance goals. For athletes seeking acute performance enhancement, liquid formulations of GPLC taken 30–60 min pre-exercise with a small carbohydrate source optimize absorption and bioavailability. For individuals focused on recovery and structural adaptation, divided daily dosing with emphasis on post-exercise administration proves most effective. Combination approaches integrating glycine supplementation with protein and carbohydrate intake post-exercise demonstrate synergistic effects on muscle protein synthesis and glycogen replenishment. Practical implementation should consider individual factors including training status, age, health conditions, and specific performance objectives, with particular attention required for individuals with renal impairment due to glycine's metabolic pathways.

Despite promising evidence, several research gaps necessitate attention. The field lacks long-term safety data extending beyond 12 weeks, creating uncertainty about chronic supplementation implications. Mechanistic studies in human models remain limited, with most molecular insights derived from preclinical investigations. Standardization of exercise protocols across supplementation studies would enhance comparability and evidence synthesis. Future research should prioritize population-specific investigations, particularly in older adults and female athletes who remain underrepresented in current literature. Advanced imaging techniques and molecular analyses could provide deeper insights into glycine's tissue-specific effects and temporal dynamics during different exercise modalities. In practical application, glycine supplementation represents a promising nutritional strategy with multiple mechanisms supporting exercise performance and recovery. The evidence supports its integration into comprehensive athletic nutrition programs, particularly for individuals engaged in high-intensity or prolonged exercise regimens. However, implementation should be guided by individual assessment, considering both the potential benefits and the current limitations in evidence regarding long-term use and population-specific effects. As research continues to evolve, glycine and its derivatives may assume an increasingly prominent role in evidence-based sports nutrition, bridging the gap between metabolic support, structural integrity, and performance optimization in athletic populations across the lifespan.
